# Research on the Influencing Mechanism of Paradoxical Leadership on Unethical Pro-Supervisor Behavior

**DOI:** 10.3390/bs12070231

**Published:** 2022-07-14

**Authors:** Suchao He, Xiaoying Yun

**Affiliations:** 1 The Business School, Northwest Normal University, Lanzhou 730070, China; hsciswufe@nwnu.edu.cn; 2 School of Economics and Management, Chuzhou University, Chuzhou 239000, China

**Keywords:** paradoxical leadership, supervisor–subordinate Guanxi, follower mindfulness, unethical pro-supervisor behavior

## Abstract

Paradoxical leadership is a leadership style that combines both employees’ individual needs and organizational requirements. The existing literature shows that paradoxical leadership has a positive influence on variables at the individual level, team level and organizational level. It is necessary to further explore the negative impact of paradoxical leadership on the individual level (such as employees’ unethical pro-supervisor behavior), the path of influence and situational conditions. Based on social exchange theory, this paper studied the influence of paradoxical leadership on employees’ unethical pro-supervisor behavior, and clarified the mediating role of supervisor–subordinate Guanxi and the moderating effect of follower mindfulness. We conducted an empirical analysis on the data of 356 employees collected in two phases, and found that paradoxical leadership exerts a significant positive effect on unethical pro-supervisor behavior; supervisor–subordinate Guanxi has a partial mediating effect on the relationship between paradoxical leadership and unethical pro-supervisor behavior; and follower mindfulness moderates the influence of paradoxical leadership on supervisor–subordinate Guanxi, and moderates the intermediation of supervisor–subordinate Guanxi on the main effect. This paper enriches the existing research on the mechanism of influence of paradoxical leadership and deepens our understanding of boundary conditions in relation to the role of paradoxical leadership.

## 1. Introduction

In the late 1990s, the United States put forward the concept of VUCA (volatility, uncertainty, complexity, and ambiguity) in military management [1], indicating that the world is facing a paradigm shift in the information age. With the development of the times, leaders in organizations need to develop specific strategies for action in the face of many uncertainties, and VUCA has become shorthand for the turbulent situations leaders may encounter. VUCA (volatility, uncertainty, complexity, and ambiguity) is used to describe executives’ perception of the environment as difficult to confidently diagnose and extremely confusing.

During times of VUCA (volatile, uncertain, complex and ambiguous) circumstances, an organization faces many contradictions and paradoxes during the process of operation and development [2]. In order to choose a path through the fog, leaders need organizational skills that can deal with insufficient insight, foresight, and broad understanding to prepare for many types of upheaval [3]. The manager’s ways of effectively dealing with the organizational contradictions and paradoxes will determine the organization’s future [4]. However, previous “either−or” leadership has found it difficult to effectively address the contradictions and paradoxes [5]. Therefore, Zhang, Waldman, and Han (2015) combined the traditional philosophy of yin and yang with leadership theory in the West and proposed paradoxical leadership (PL), a leadership style which meets both the requirement of organizational structure and employees’ needs (these requirements compete and correlate with each other) [6]. PL highlights “both−and” logic and the displaying of leadership in a complicated and dynamic environment [7], so as to effectively resolve contradictions and paradoxes in the organization.

At present, the research on paradoxical leadership mainly focuses on its impact on individuals, teams, and organizations. At the individual level, Zhang, Waldman, and Han (2015) demonstrated that paradoxical leadership positively affects employees’ job proficiency behavior, adaptive behavior, and proactive behavior [6]; Yang, Li, and Liang (2021), using survey data from a 139 leader-employee paired sample of Chinese companies, show that paradoxical leadership positively affects employee creativity through the mediating effect of job prosperity, while enhancing psychological safety found a positive relationship between job prosperity and employee creativity [8]; Li, Yan, and Wang (2018), based on social exchange and social information processing theory, tested the idea that paradoxical leadership has a significant positive impact on employees’ facilitative and inhibitory voice behaviors, and found that moderation focus moderated psychological safety in paradoxical leadership and voice behavior mediation between them [9].

At the team level, researchers believed PL can positively improve team cognition [10]. Peng and Li (2018) found that when paradoxical leadership prevails, teams with diversity of expertise show better innovation performance [11]; Luo, Hua, and Zhong (2015) found the paradoxical leadership can play a positive role in team innovation, and knowledge creation and knowledge integration can play a full mediating role in it [12].

At the organizational level, paradoxical leadership has a positive impact on the organization’s ambidextrous innovation ability by influencing knowledge sharing [13]. Some scholars mainly found PL had an active effect on the organizational creativity [14], strategy paradox [15], and organizational competitive edge [16].

Most of these studies focused on positive effects. Negative effects will emerge if the leader is not competent in PL, employees fail to adapt themselves to PL, or the leader and employees have difficulties in meeting work requirements with the help of existing work resources [17]. However, we know very little about the “dark side” (such as unethical pro-supervisor behavior) of paradoxical leadership, and there is a need to further explore this [8]. Additionally, we do not know whether paradoxical leadership has an impact on unethical pro-leadership behavior, and what the mechanism of a potential impact may be. Are there any boundary conditions? Further studies need to be conducted to answer the above questions [17,18].

UPSB refers to employees’ extra-role behavior based on unethical pro-supervisor behavior, which has two characteristics: “pro-supervisor” and “unethical” [19]. In an organization, leaders control resources and have the right to make decisions. Pro-supervisor behavior is the active and conscious behavior of employees; the motivation for unethical behavior is to benefit leaders, who then help individuals with unethical behaviors to gain benefits [20].

In the past, studies on the influence of unethical pro-organizational behavior were conducted from the perspective of organizational factors, leadership style, and individual factors [21,22]. These studies were mostly based on Western situations and focused on the single leadership style. Moreover, there has been insufficient in-depth analysis on the formation mechanism of UPSB in China [23]. It is essential to study the influence of other leadership styles (such as PL) on unethical pro-organizational behavior [24]. Highlighting the “both-and” logic, PL matches the “pro-supervisor” and “unethical” characteristics of UPSB.

In addition, in Chinese enterprises characterized by relational orientation and authoritarian orientation, the supervisor−subordinate Guanxi based on Chinese culture can have a significant impact on subordinate behaviors (especially pro-leadership behaviors) [21]. Leadership is a direct factor influencing how employees to perceive the work environment. Compared with other leadership styles, the paradoxical leadership style breaks through the dilemma of “choose one of two” and displays behavioral abilities that seem to be competitive but are actually closely related [6], which can influence the unethical behavior of subordinates and relatives through the relationship between superiors and subordinates, and uncover the “mechanism black box” of the influence of paradoxical leadership on the unethical behavior of subordinates and relatives.

Therefore, based on the social exchange theory, this paper, focusing on Chinese cultural situations, probes the mechanism of influence of PL on UPSB and specifies the intermediate role of supervisor−subordinate Guanxi (SSG); and the moderating effect of follower mindfulness (FM). This paper is expected to be able to provide answers to the above questions and to scientifically evaluate and guide employees’ UPSB.

## 2. Theoretical Basis and Research Hypotheses

### 2.1. Paradoxical Leadership (PL)

In an increasingly complex and volatile competitive environment, organizations inevitably face various conflicts, such as the conflict between implementing change and maintaining stability [25], and the conflict between short-term profitability and long-term sustainable development [26]. These seemingly contradictory needs are actually interdependent; this phenomenon is known as a “paradox” [7], and the conflicts and paradoxes have become the “new normal” in the current uncertain organizational environment [3]. Therefore, in the context of environmental uncertainty, how leaders effectively deal with the challenges brought about by paradoxes is crucial to the survival and development of organizations [27]. To better cope with the challenges posed by paradox, leaders need to play multiple contradictory roles and adopt paradoxical behaviors [28].Therefore, combining the paradox perspective with leadership research, Zhang, Waldman, and Han (2015) put forward the concept of paradoxical leadership (PL) based on the Chinese yin and yang philosophy [6].

To effectively meet organizational requirements and employees’ needs which seem to be contradictory but interrelated, leaders are required to play several contradictory roles and to implement contradictory behaviors [27]. Based on the organizational paradox and Oriental yin−yang philosophy, Zhang et al. (2015) [6] proposed “paradoxical leadership” (also known as “contradictory leadership”), a leadership style that meets organizational requirements and employees’ needs, which seem to be competitive but interrelated. PL describes “both−and” characteristics from five dimensions: egotism and other-orientation combination, being intimate and keeping at arm’s length, treating subordinates equally without discrimination and allowing individuation, having a strict work requirement and maintaining flexibility, and upholding decision control and allowing independence [6]. PL breaks through single situational leadership’s limitation on time and space and improves synergy with overall thinking with the help of paradoxical thinking [29], achieving the effectiveness of leadership.

Paradoxical leadership (PL) refers to leaders adopting seemingly competitive but interrelated behaviors designed to simultaneously satisfy competing demands at work [6]. Based on the dual meanings of meeting the structural needs of the organization and meeting the individual needs of employees, Zhang et al. (2015) propose five dimensions of paradoxical leadership: (1) combining self-centeredness with other-centeredness (SO); (2) maintaining both distance and closeness (DC); (3) treating subordinates uniformly, while allowing individualization (UI); (4) enforcing work requirements, while allowing flexibility (RF); (5) maintaining decision control, while allowing autonomy (CA) [6]. For example, an organization needs to continuously improve existing products and develop new products; employees are expected to work independently and strengthen the teamwork; managers are required to be more authoritative and to strengthen control. In the face of these complex paradoxical issues, the traditional ether or management method will cause the organization trouble, while the paradoxical leadership that emphasizes the harmonious coexistence of opposing elements can be fully realized.

### 2.2. Paradoxical Leadership and Unethical Pro-Supervisor Behavior

Unethical behavior is behavior of organizational members which violates moral criteria and social norms [20]. Organizational members may exhibit unethical behaviors for their benefit, revenge, or altruism [20,30]. Unethical pro-supervisor behavior is derived from unethical pro-organizational behavior. Johnson and Bingham (2011) define unethical pro-organizational behavior as a violation of the behavior of core social values, laws and regulations, public order, and good morals or moral standards aiming to promote the effective operation of the organization as a whole or its members (e.g., leaders) [31]. Therefore, unethical pro-supervisor behavior includes two components: pro-organizational motivation and unethical behavior. Johnson and Umphress (2019) expanded the research on unethical pro-organizational behavior, pointing out that employees will not only display unethical behaviors for the benefit of the organization, and possibly make unethical behavior for the benefit of leadership [32]. Such behaviors that aim to safeguard the interests of leaders but violate the core values of society, laws and regulations, and public order and good morals, are called unethical pro-supervisor behavior (UPSB). Mesdaghinia, Lewis, and Eisenberger (2019) found that the biggest difference between unethical pro-organizational behavior and unethical pro-supervisor behavior is that the former’s behavioral purpose is to benefit the organization, while the latter’ s focus is much narrower, mainly serving the leader’ s interests, even at the expense of the organization for the benefit of the leader [33].

Employees may engage in unethical pro-supervisor behavior for a variety of motivations. For example, employees may indirectly benefit from these behaviors by making their leaders happy, or they may genuinely care about their leaders, or see their leaders’ success as their own. Unethical pro-supervisor behavior includes both action and inaction. When taking unethical actions as a way of doing things, employees may engage in unethical acts of commission to help their supervisors, such as lying to protect their supervisors, misrepresenting information to beautify their supervisors, or exaggerating their supervisors’ performance for the benefit of the supervisor; or employees may engage in unethical acts of omission, such as withholding information that could damage the supervisor’s reputation.

On the basis of meeting the leader’s requirement, the paradoxical leader takes into consideration employee demands and personal ability, makes appropriate authorizations, integrates employee demands with those of the leaders, and achieves dynamic balance from a long-term perspective [27,34]. According to different situations, the paradoxical leader has a close and friendly relationship with employees but keeps them at arm’s length, takes care of employees’ personal life and organizational demands, and achieves equal organizational hierarchy with employees by integrating and resolving contradictions ([35], which improves employees’ recognition of the leader and gratitude to the organization [32]. Employees’ recognition of the organization and leader results in their UPSB. Employees identified by top leaders have a strong motivation to help the leader, leading them to withhold information potentially harmful to the leader and engage in similar unethical behavior to protect and support the leader [32]. PL leads to negative effects such as UPSB if the existing work resources are unable to realize and meet high work requirements or if employees find it difficult to effectively adapt to PL [17]. Yan and Zeng (2018) believe that transformational leaders ignore the impact of social norms on social interests in behavioral decisions, leading to leaders who may violate social ethics and laws and regulations to seek solutions to problems. Meanwhile, employees believe that helping leaders will create benefits for the organization, and thus, choose to engage in pro-leadership unethical behavior [36]. Zhong and Wang (2019) believe that self-sacrificing leaders are willing to make sacrifices for employees and teams to help others gain benefits, who then give feedback to the leader. The stronger the motivation for feedback, the more likely subordinates are to challenge the moral bottom line and implement unethical pro-supervisor behavior [37].

According to social exchange theory, the essence of the communication between individuals in an organization is a series of exchanges based on the “reciprocity principle” [38]. Based on the “reciprocity principle”, one party in the relationship needs to undertake the obligation to repay the “vested interest”, while also obtaining the “vested interest”. When one party provides the resources needed by the other party, the other party will reciprocate; when one party’s resources are damaged, it will retaliate against the other party to achieve a balance. The size of the reward motivation is affected by the quality of the interaction between the two parties. When the other party satisfies people’s needs, their reward motivation will increase; otherwise, the reward motivation will decrease.

Based on this, employees will generate a relatively strong sense of feedback and sense of mission towards the paradoxical leader during the work process to increase the “pressure of reward” on the leader. If employees have a relatively strong motive to show gratefulness to the leader and behave in the name of the leader’s benefits, they will violate moral criteria to meet the leader’s needs and implement unethical behavior [39]. During the process of implementing unethical behavior, in fact, employees may think that upholding the leader’s benefits is protecting the organizational benefits, such that they view UPSB as one way to make a contribution to the organization and use it to provide an explanation for their unethical behavior [40]. Therefore, employees rationalize pro-leadership unethical behavior, implement moral shirking, and reduce moral guilt to implement pro-leadership unethical behavior. Furthermore, employees may implement UPSB based on the trust of their reciprocal relationship with the leader in order to obtain rewards from the leader. They acquire profitable economic exchanges by forming a transactional psychological contract relationship with the leader. Fundamentally, they behave this way for their own sake [41].

In conclusion, this paper proposes the following hypothesis:

**H1.**  *PL is positively correlated with employees’ UPSB.*

### 2.3. Intermediating Effect of SSG

The concept of Guanxi has been interpreted by many sociologists and psychologists since Fei Xiaotong proposed the “differential order pattern” in China [42]. Guanxi, also known as interpersonal relationship, refers to the special connection between people and is the core concept of all social strata [43]. In the context of Chinese organizations characterized by “power distance” and “guanxi orientation”, Guanxi is regarded as a local variable in China [44]. Supervisor−subordinate Guanxi (SSG) refers to the special relationship between the superior and subordinate based on emotion and status. It directly affects the psychological states and behavioral expectations of both parties in the subsequent contacts [45]. SSG not only includes contact during work, but also is carried out through activities outside work, such as social gatherings, gifts, and visits [46]. This relationship is rooted in the common interests and hobbies of both parties. It is the interaction between leaders and subordinates; that is, it depicts the informal and special social connections between superiors and subordinates outside the work field. Based on extensive interaction, this article focuses on subordinate and subordinate relationships in personal workplace relationships [47]. The situational factor cannot be ignored in the research on the influence of leadership style on employee behavior, while SSG is an important situational factor [45].

In the context of Chinese culture, the relationship between superiors and subordinates is rooted in the mutual understanding of both parties. Interests and hobbies allow interaction between leaders and subordinates, and describe the informal and specific social connections established by leaders and subordinates outside the workplace. As employees are in direct touch with the leader during the work process, the close SSG not only helps employees obtain more resources from the leader and achieve promotion, but also facilitates the leader in implementing unethical behavior with the help of employees. Supervisor−subordinate Guanxi more accurately describes the core characteristics of the relationship between superiors and subordinates in Chinese organizations (such as interpersonal contacts beyond the scope of work, preferential treatment based on particularism, etc.), and has a greater influence on subordinates’ behaviors (especially pro-leadership behaviors). In “relation orientation” and “authority orientation” of Chinese society, the relationships between superiors and subordinates are more sensitive than in the West, require more attention, and have more influence on organizational members’ behavior [48], and therefore in the context of Chinese culture, the relevant stakeholders in the organization studying relationships between superiors and subordinates obviously has more practical significance.

A paradoxical leader can treat their subordinates equally and make equitable and reasonable decisions [6,49]. As a result, employees have a strong perception of the leader’s fairness and are willing to construct a sound SSG. Liu, Long and Li (2003) [50] found that a disinterested leader has a significant predictive effect on positive outcomes, and the leader’s prejudice is reduced when fairness is perceived by employees, leading to high-quality SSG. Moreover, paradoxical leaders “combine egotism with others’ orientation”, balance organizational structural demands and employee individual demands, and help employees reach an equilibrium between work and family [51], which promotes the construction of a favorable SSG. Furthermore, paradoxical leaders allow employees to have job autonomy, maintain job flexibility, and satisfy employees’ demands for self-fulfillment [8]. This achieves leader–employee interaction and cooperation such that employees maintain positive mental elasticity and maintain a close relationship with their leaders [12]. Paradoxical leaders are able to effectively combine a sense of distance with a sense of intimacy and maintain relationships with subordinates in a status-based, hierarchical, and trust-oriented manner [29], which advances employees’ perception of the leaders’ insider status and strengthens their relationship.

An SSG is formed by the connection of interests, emotion, and status [38]. According to social exchange theory, in order to obtain resources allocated by the leader, employees are willing to actively pay for the leader in order to build a good relationship between superiors and subordinates [52], even committing unethical actions for close leaders to achieve the goal of having “inside people”. During the construction process, employees have a prominent pro-supervisor motive and are driven to take some pro-supervisor actions to meet the leaders’ requirements, even though such actions are unethical. From the perspective of outcome, implementing UPSB can bring more benefits to the leaders and employees [53] and lead to more behaviors such as these.

Social exchange theory holds that if one party to the exchange provides a benefit to the other party, the recipient will form a willingness to reciprocate [45]. Accordingly, this study believes that, based on the logic of “reward”, pro-leadership behavior reflects the subordinate’s repayment obligation to the leader. Subordinates can obtain better welfare and care from high-quality superior–subordinate Guanxi, so that subordinates have a stronger sense of obligation to return [6], and show more pro-leadership behaviors. However, due to the difference in the status of leaders and employees, there is unfair interaction. This inequality causes subordinates to maintain leadership interests at any cost in high-quality superior–subordinate Guanxi, resulting in subordinates showing more pro-supervisor and unethical behaviors.

According to social exchange theory, the nature of relationships is resource exchange, and such resource exchanges construct the basis of relationship quality [54]. In addition to work interactions, SSG also involves social interactions and personal relationships [55]. Paradoxical leaders have a vague boundary of work and family. When they construct a familistic high-quality relationship with their subordinates, the supervisors provide more resources to the subordinates, such as information, resources, assistance, and opportunities for promotion. Based on the principle of reciprocity, the trust and obedience to leaders of subordinates are enhanced when they construct a high-quality relationship with leaders [56]. In such situations, employees, out of a sense of work responsibility and the reciprocal obligation of generating returns for leaders (in other words, employees have sufficient pro-supervisor motives), believe that they belong to the leaders’ “inside circle”. As a result, they will lower their moral standard, confuse moral awareness, and are willing to undertake risks and implement uncertain and high-risk extra-role behavior (that is, UPSB) for the sake of leaders [57]. According to this, taking SSG in Chinese situations as a mediating variable, this paper proposes the following hypothesis:

**H2**  **.***SSG has a mediating effect on the relationship between PL and employees’ UPSB*.

### 2.4. Moderating Effect of FM

Mindfulness originated from Eastern Buddhist philosophy. In the 1970s and 1980s, Kabat-Zinn (2005) introduced mindfulness into the field of organizational management, believing that mindfulness is a way to focus on the present with purpose and without judgment [58]. Employees with a high level of mindfulness are able to focus on the present moment and quickly disengage from negative events while maintaining a keen awareness; employees with a low level of mindfulness are easily immersed in past or future emotional events, causing them to fall under the influence of negative events.

In an organization, employees have different levels of sensitivity and processing ability; these individual traits determine the differences in leader–employee relationships [59]. Among the individual traits, FM is considered as a stable functional trait for individuals to confront internal and external stimuli. FM refers to the individual trait of attention and awareness which allows an employee to focus on current acceptable situations and have no judgment [60]. High FM can strengthen the relationship between leader and employee behaviors. Eisenbeiss and Knippenberg (2015) [61] found that a higher level of FM led to a stronger relationship between moral leadership and proactive employee behaviors. Zhang, Song, Zheng, and Ni (2018) [62] found that the interaction between the trait mindfulness of leaders and subordinates can promote leader–member relationships and enhance employees’ work input. Therefore, we believe that FM can have a significant effect on PL and employees’ UPSB.

Mindfulness includes the self-regulation of attention and current specific guiding experiences [63]. Employees who have high FM display sound self-regulation of attention and are good at noticing and observing negative organizational experiences [62]. They are able to acutely perceive leaders’ care and respect, separate internal experience from external stimulus with the help of self-regulation, and reduce internal resource losses so as to enhance mutual understanding and trust, which is beneficial to constructing high-quality SSG [64]. Furthermore, employees who have high FM are able to maintain high focus, are consistently conscious about current situations, are attentive, devote themselves to pro-supervisor behavior, accept leaders’ orders without judgment, and do not deliberately control or avoid situations [65]. They focus attention on the resource support of paradoxical leaders and an organizational environment featuring openness and support, and build sound exchange relationships with leaders. Through the empirical analysis of leader–employee matched sample data, Zhang, Song, Zheng, and Ni (2018) [62] explored the mediating effect of leaders’ trait mindfulness on employees’ trait mindfulness and leader–member exchange relationships. From a theoretical perspective, Shen, Yang, Hu, He, and Li (2020) [66] studied the regulating effect of mindfulness (FM, state mindfulness and mindfulness training) on the relationship between abusive leaders and employee behavior (employees’ negative emotional reaction, deviant behavior and performance). Employees with high mindfulness experience less negative emotions such as psychological tension and hostility, and less retaliation against their superiors and withdrawal from work when they are subjected to abusive treatment by their superiors.

**H3.**  *FM has a positive moderating effect on the relationship between PL and SSG; that is, high FM can strengthen the relationship between PL and SSG.*

Based on H1 and H3, this paper proposes a moderated mediation model, considering the mediating effect of SSG on PL and employees’ UPSB changes along with the change in FM. Compared with employees who have low FM, employees who have high FM are able to self-regulate and respond to internal and external stimulus with a neutral attitude. They are willing to accept new things and have a more harmonious relationship with their subordinates. Based on the principle of reciprocity in social exchange theory and the spirit of the contract in supervisor–subordinate resource exchange, employees generate a sense of obligation to generate reward and highlight a “pro-supervisor” attribute while neglecting “unethical” attributes. They believe that implementing UPSB is a favor offered by leaders, and so they should make returns to the leaders. As a result, they feel free to undertake risks and implement UPSB. On the contrary, employees who have low FM lack attention and devotion. Therefore, they display a weaker obligation in terms of reciprocity and returns, and are less likely to have a pro-supervisor motive and to demonstrate unethical behavior.

Combining Hypothesis 2 and Hypothesis 3, this paper proposes a moderated mediation hypothesis model: The mediating effect of the superior–subordinate Guanxi on the relationship between paradoxical leadership and employees’ unethical pro-supervisor behavior will change due to the change in employee trait mindfulness. Compared with employees with low trait mindfulness, employees with high trait mindfulness are able to self-regulate, actively respond to internal and external stimuli, are willing to accept new things, and have a more harmonious relationship with their subordinates. In order to maintain this high-quality subordinate relationship, employees will make full use of their abilities to protect the interests of leaders, lower their own moral thresholds, violate social morality and laws in order to achieve their goals, and are more willing to show unethical behavior for leaders. Schultz and Ryan (2015) verified the moderating effect of employee trait mindfulness, and found that mindfulness positively moderated the positive relationship between a self-supporting work atmosphere and employees’ job well-being [67]. Liang et al. (2014) also verified that leadership trait mindfulness can reduce the hostility of abusive leaders to their subordinates [68]. When leaders with high trait mindfulness perceive hostility, their self-mindfulness adjustment ability is stronger, and it is easier for them to adjust their moral benchmarks. Thus, similarly, employees with higher trait mindfulness may engage in unethical behaviors due to their closeness to their leaders.

Based on this, this paper proposes the following assumptions:

**H4.**  *FM moderates the mediating effect of SSG on the relationship between PL and employees’ UPSB*.

**Figure 1:**
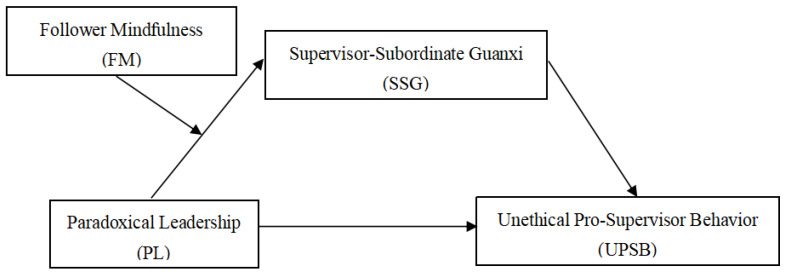
Theoretical framework.

According to social exchange theory, the mechanism of influence of SSG on PL and UPSB is a top-down process of resource transfer. Through the internalization process of “leadership style”−“SSG” −“employee behavior”, this paper constructs a moderated mediation model to explain the internal mechanism of employees’ unethical behavior. The mechanism of influence of PL on UPSB includes four paths: (1) PL directly influences UPSB; (2) PL affects UPSB through SSG; (3) FM moderates the relationship between PL and SSG; and (4) FM moderates the indirect effect of SSG on the relationship between PL and UPSB (FM moderates the relationship of PL >>> SSG >>> UPSB). The theoretical framework of this research is shown in Figure 1.

## 3. Research Design and Methods

### 3.1. Research Sample

The samples in this study are all from four provinces in China: Gansu, Shanxi, Anhui, and Yunnan. The industries of the sample distribution include internet, manufacturing, banking, and real estate. Before our formal research and survey, we conducted interviews with the leaders of five manufacturing companies in Gansu Province and determined the scientific nature of the research questions and the reasonability of the questionnaires. In the process of distributing and collecting the questionnaires, we first approached the company manager, human resources department, and other relevant departments, and distributed the questionnaires among the employees by sending links. The employees filled in the questionnaires independently within a specified time, and the questionnaires were submitted anonymously. In order to ensure the authenticity of data collection, the instructions at the beginning of the questionnaire clearly indicated that the survey results were only for academic research and did not involve any commercial use or privacy issues. The survey was completely anonymous, and the survey results strictly confidential.

During the survey process, a two-phase data collection method was used to reduce the likelihood of possible homologous mistakes. Firstly, we distributed 400 questionnaires on PL and UPSB, with 389 valid questionnaires being returned. Two months later, we distributed questionnaires on SSG, FM, and employee power distance orientation and collected 356 valid questionnaire responses. The specific demographic variables are shown in Table 1.

As can be seen from Table 1, among the respondents, there were 172 males, accounting for 48.3%, and 184 females, accounting for 51.7%; in terms of age, there were 114 respondents aged 25 and below, accounting for 32%, 135 employees aged 26–35, accounting for 397.%, 54 employees aged 36–45, accounting for 15.2%, and 53 employees aged 46 and above, accounting for 14.9%; in terms of education level, most of the respondents had a college or undergraduate degree, accounting for approximately two-thirds of the total number of samples; in terms of the length of employment of the respondents, 15.4% of the respondents had been employed for 1–6 months, and 17.4% had been employed for 6 months to 1 year, 22.5% had been employed for 1 year to 2 years, and 44.7% had been employed for more than 2 years; in terms of tenure, the respondents who served for 1 to 2 years accounted for 22.5%, and for more than 2 years, 159 people, accounting for 44.7%; in terms of job types, 83.7% of the employees surveyed were ordinary employees and grassroots managers; and in the nature of enterprises, private enterprises accounted for 61.5%.

### 3.2. Measuring Tools

The measurement scales in this paper are frequently used by overseas researchers because of their high reliability and validity. According to the procedures of double-blind translation and retranslation, the scales were translated into Chinese and some questions in the scales were modified appropriately according to the interview conducted before the survey. Five-point Likert scoring was used in the scales, where “1” referred to “strongly disagree” and “5” referred to “totally agree”.

(1) PL: The scale developed by Zhang et al. (2015) [6] was used, which included 22 items, such as, “My leader treats all subordinates without discrimination but also takes into account their personal characteristics”, “My leader has prestige as a leader but also shares the leadership role with subordinates”. The Cronbach’s α of this scale is 0.902.
(2) SSG: The SSG scale developed by Law et al. (2000) [45] was used, which included six items such as, “My leader may invite me to his/her home for dinner”, and “I will visit and give a gift to my leader at special festivals (such as the leader’s birthday)”. The Cronbach’s α of this scale is 0.853.
(3) UPSB: The scale developed by Johnson and Umphress (2019) [32] was used, which included six items such as, “If necessary, I may cover up the information which may be bad for my leader”, and “I exaggerate my leader’s performance because it can help my leader”. The Cronbach’s α of this scale is 0.855.
(4) FM: The Mindful Attention Awareness Scale (MAAS) developed by Brown and Ryan (2003) [60] was used, which included 15 items such as, “I could be experiencing some emotion and not be conscious of it until some time later”, and “I find it difficult to stay focused on what’s happening in the present.”. The Cronbach’s α of this scale is 0.857.
(5) Control variables: The employees’ individual characteristics (gender, age, education background, and years of work) and company traits (the nature of company) may exert influence on employees’ UPSB. Masuda and Nisbett (2001) [69] found that employees’ power distance orientation may affect structure and subordination. Therefore, we controlled employees’ individual characteristics (gender, age, education background, and years of work), company traits (the nature of company) and employees’ power distance orientation. The questionnaires concerning employees’ power distance orientation were developed by Howell et al. (1986) [59], and included six items, such as, “My leader does not need to ask for my opinion when making decisions”.

## 4. Results

### 4.1. Common Method Bias and Confirmatory Factor Analysis

The sample data of this research were obtained from the questionnaires filled in by respondents. A two-phase data collection method (lasting about three months) was used to reduce possible the occurrence of homologous mistakes. Harman’s single-factor test showed that the variance-explained ratio of the first principal component was 25.84%, which was much lower than the boundary level (50%).

This indicated that there was no significant common method bias in this research.

The confirmatory factor analysis method was used to test the effectiveness of latent variables. We used mplus8.0 for confirmatory factor analysis and found that, compared with three-factor, two-factor and single-factor models, the four-factor model had the best fitting indexes, as shown in Table 2. The ratio of chi-square to degrees of freedom was 1.986; IFI = 0.903, CFI = 0.903, NFI= 0.822, NNFI = 0.898, RMSEA = 0.053, and RMR = 0.091, Although according to the requirements of Hu and Bentler (1999) [70], the standard of CFI and NFI is not less than 0.90. However, in the paper of Tao, Wu, and Hu (2022) [71], when they discussing the influence of paradoxical leadership on employee creativity. The result of confirmatory factor analysis shows TLI = 0.88, NFI = 0.82, the index value is considered acceptable, and further research can be carried out. Confirmatory factor analysis results in the paper “Creativity under workload pressure and integrative complexity: The double edged sword of paradoxical leadership” by Shao, Nijstad, and Täuber (2019) in *Organizational Behavior and Human Decision Processes* showed that TLI = 0.87, CFI = 0.88 [72]. Therefore, according to the research of previous scholars, we believe that the values of NFI and NNFI in the paper basically meet the requirements, and further research can be carried out. The index reached the optimal state. This suggested that the four variables were well distinguished.

### 4.2. Descriptive Statistics and Correlation Analysis

SPSS 22.0 software was used to conduct descriptive statistical analysis and analysis of four core variables: PL, UPSB, SSG, and FM. Later, correlation analysis was conducted concerning the relationships between PL and UPSB, between PL and SSG, between SSG and UPSB, and between FM and UPSB.

According to Table 3, PL has a significant positive relationship on UPSB (r = 291, *p* < 0.01); PL has a significant positive relationship on SSG (r = 0.395, *p* < 0.01); SSG has a significant positive relationship on UPSB (r = 0.228, *p* < 0.01); and FM has a significant positive relationship on UPSB (r = 0.076, *p* < 0.05).

### 4.3. Hypothesis Test

SPSS 22.0 software and the bootstrap method were used to test the mediating effect [64]. A 95% confidence interval was obtained after 5000 bootstrap samples. The test results of the hierarchical regression analysis method are shown in Table 4. According to these results, PL can significantly promote employees’ UPSB (β = 0.341, *p* ≤ 0.001), which indicates that a higher level of PL leads to a greater possibility of employees demonstrating UPSB. Therefore, H1 is verified. After adding the mediating variable of SSG, PL still has a significant direct predictive effect on UPSB (β = 0.280, *p* ≤ 0.001); PL has a significant positive effect on SSG (β = 0.407, *p* ≤ 0.001); and SSG also has a significant positive predictive effect on UPSB (β = 0.151, *p* ≤ 0.001). Moreover, after adding SSG, the influence level of PL on employees’ UPSB decreases from 0.341 to 0.280. Therefore, SSG partially mediates the relationship between PL and UPSB. As a result, H2 is verified.

Based on the plug-in PROCESS in SPSS 22.0 software, the bootstrap method was used to conduct 5000 tests to further verify the mediating effect of SSG. The results show that the direct effect of PL on UPSB remained within the 95% confidence interval (0.151, 0.409). The test results of the bootstrap method show that the 95% confidence interval (0.307, 0.507) does not include 0, which indicates that PL can not only directly predict UPSB, but also predict UPSB through the mediating effect of SSG. The direct effect and mediating effect account for 82.1% and 17.9% of the total effect, respectively, as shown in Table 5. Consequently, H1 and H2 are further verified.

The moderating effect of FM is shown in Table 5. After FM, the interaction items of PL and FM are added into the model, which both have a significant predictive effect on SSG (β = 0.099, *p* < 0.05), suggesting that FM can moderate the effect of PL on SSG.

**Figure 2:**
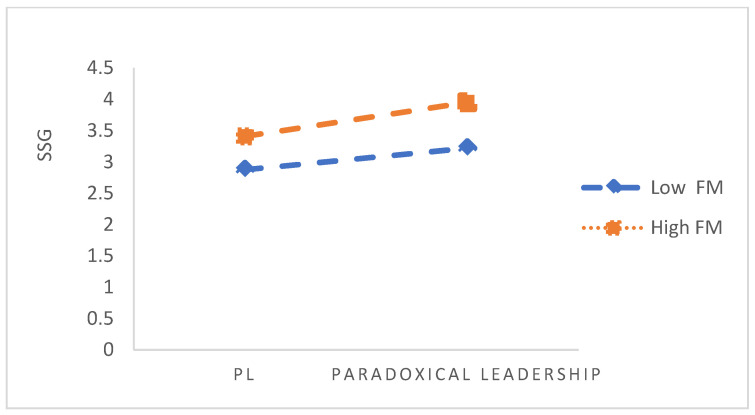
Moderating effect of FM on PL and SSG.

In order to further verify the moderating effect of FM, a moderating effect graph was constructed, which is shown in Figure 2. According to the graph, we found that compared with the low levels of FM (M − SD), high levels of FM (M + SD) can strengthen the relationship between PL and SSG. PL has a stronger positive effect on SSG when employees have high FM (M + SD). It can be seen from the figure that, compared with individuals with FM (M − SD), in individuals with high mindfulness, paradoxical leadership can significantly positively affect the relationship between superiors and subordinates. Therefore, H3 is verified.

The PROCESS plug-in macro-program in the SPSS 22.0 software was used to test the moderated mediating effect, calculating the mediating effect at different FM levels. According to Table 6, in the lower-level group of FM (M − SD), the indirect effect is not significant (95% confidence interval contained 0, (−0.01,0.173)); however, in the higher-level FM group, indirect effects is significant (95% confidence interval did not contain 0, (0.024, 0.171)). Therefore, we believe that FM can moderate the influence of PL on UPSB through SSG. As a result, H4 is verified.

## 5. Discussion and Conclusions

### 5.1. Research Conclusion

Superior−subordinate Guanxi refers to the personal relationship between superiors and subordinates, which mainly includes personal communication, emotional interaction and awareness of responsibility between superiors and subordinates outside work [73]. This paper analyzed the mechanism of influence of PL on employees’ UPSB and its boundary conditions. On the basis of social exchange theory, this research was conducted from the perspective of interpersonal interactions, featuring “leadership–SSG–employee behavior”. Based on data analysis from 356 valid questionnaires, we profoundly explored the relationship between PL and UPSB. The results show that PL has a significant positive effect on UPSB; PL partially and positively affects employees’ UPSB through the transmission route of SSG; and FM has a positive effect on the influence of PL on UPSB.

### 5.2. Research Contribution

This paper connected PL with employees’ UPSB and revealed that the leader’s paradoxical management would result in the employees’ unethical behavior for the sake of the leader. A leader is an important influencing factor in the member–organization relationship [40]. In terms of the influence of PL, other scholars have mainly focused on active employee behavior, creativity and other positive effects [6,34]. However, there has been insufficient exploration of the negative influence caused by PL [17,18], and it is therefore urgent to further explore the ‘dark side’ of paradoxical leadership. To this end, this paper studied the influence of PL on UPSB. Here, it is made clear that “grayscale management” leadership, through organizational identification, could enhance the influence of SSG on employees’ negative behavior. This research also revealed the potential dark side of PL, in agreement with the research conducted by Wang, Long, and Peng [74], who proposed that positive leadership has an influence on negative behavior, which enriches the negative outcomes of paradoxical leadership to a certain extent.

On the basis of social exchange theory and with a focus on Chinese situations, this paper verified the functional mechanism of SSG in terms of PL and employees’ UPSB. Running through daily organizational operation, SSG facilitates two-way communication and resource access and affects employee behavior, personal friendships between employees and leaders, and other important situations [39]. Therefore, it is necessary to combine this research with Chinese situations and to take into account the influence and effect of SSG on PL and UPSB. Starting from actual situations of Chinese companies, this paper explored the mediating mechanism of SSG in the relationship between PL and UPSB, deepened the understanding of the mechanism of influence of SSG, demonstrated the possible negative behaviors caused by SSG, and provided an empirical basis for relationship studies in Chinese situations.

Last but not least, this research introduced an individual characteristic of employees FM to explore the boundary condition of PL and SSG. Previous studies found that, due to differences in individual characteristics, not all individuals could adapt themselves to and benefit from PL [6,34]. Therefore, this study verifies the boundary effect of individual personality traits between the superior subordinate relationship and unethical pro-supervisor behavior, and introduces the variable of employee trait mindfulness into the leader–employee interaction, which expands the research on the boundary conditions of unethical pro-supervisor behavior. This research found that employees who had high FM, affected by PL, were able to develop sound personal relationships with supervisors and to better obtain resources concerning both work and family. Out of the principle of reciprocity and in the spirit of the contract, employees are willing to implement UPSB for the sake of their leaders.

### 5.3. Management Enlightenment

In an era of VUCA circumstances, companies are facing more complicated and dynamic environments. They cannot adapt to organizational needs by purely depending on previous paths and methods. It is a matter of urgency for companies to train leaders in paradoxical thinking, strengthen the leaders’ cognition of conflict and compatibility during corporate operation, and improve the leaders’ “both−and” paradoxical abilities so that they can effectively address contradictions and problems. Firstly, companies should realize the importance of organizational members’ moral behaviors and take some measures, such as internal training or cultural development, to improve the moral level of leaders and employees. In recruitment and promotion, moral standard may be regarded as an important indicator in raising the moral level of employees. Secondly, efforts should be made to enhance supervision and to support the role of leaders. The construction of enterprise systems and supervision should be enhanced to avoid UPSB. Leaders should establish correct ethics and treat their relationships with subordinates appropriately, weakening the influence of “relationships”, “friend circles”, and “human feelings”, and putting an end to employees’ unethical behavior due to sound SSG. Thirdly, mindfulness, as a state-like personal trait, can be changed through organizational changes. Therefore, managers should pay attention to the mindfulness level of employees. Managers can help their employees to improve through external interventions such as mindfulness training and daily training in the organization. The level of mindfulness can form a positive corporate culture atmosphere, so that employees can form a good working attitude to promote the development of the organization and reduce unethical behavior.

According to the social exchange theory, the essence of a relationship is a resource exchange, and the resource exchange between the two parties constitutes the basis for the quality of the relationship [75]. The relationship between superiors and subordinates brings emotions from personal relationships into organizational life, and employees who have a better relationship with their superiors can often obtain more information, resources, and promotion opportunities [56].

### 5.4. Research Limitations and Prospects

Some enlightening conclusions have been drawn in this paper, but there are some limitations: (1) A convenience sampling method was used to collect samples. (2) From the perspective of “leadership style–SSG–employee behavior”, this study only explores the unethical pro-leadership behavior produced by paradoxical leadership. Whether paradoxical leadership leads to other negative effects, such as workplace civilized behavior, employee sabotage, etc., needs further research. (3) In Chinese enterprises, it is very important to build a good relationship between superiors and subordinates. However, the relationship between superiors and subordinates in organizations is changing dynamically, and future research ought to adopt a longitudinal approach to dynamically explore the role of subordinate relationships in pro-leadership unethical behavior.

## Figures and Tables

**Table 1:** Descriptive statistics of control variables of the survey sample (*n* = 356).

Controlled Variable	Category	Quantity	Scale
Sex	Male	172	48.3%
	Female	184	51.7%
Age	25 years old and below	114	32.0%
	26–35 years old	135	37.9%
	36–45 years old	54	15.2%
	46 years old and above	53	14.9%
Educational	High school/technical secondary school and below	73	20.5%
	Junior college	124	34.8%
	Undergraduate	116	32.6%
	Master degree and above	43	12.1%
Tenures	1–6 months	55	15.4%
	6 months–1 year	62	17.4%
	1 year–2 years	80	22.5%
	More than 2 years	159	44.7%
Job type	Ordinary staff	171	48.0%
	Grassroots managers	127	35.7%
	Middle managers	47	13.2%
	Senior managers	11	3.1%
Enterprise nature	State-owned enterprises	64	18.0%
	Private enterprise	219	61.5%
	Foreign companies	32	9.0%
	Other	41	11.5%

**Table 2:** Confirmatory factor analysis result.

Model	χ^2^	df	χ^2^/df	RMSEA	RMR	IFI	CFI	NFI	NNFI
M1: PL, SSG, UPSB, FM	2322.203	1169	1.986	0.053	0.091	0.903	0.903	0.822	0.898
M2: PL + SSG, UPSB, FM	2838.547	1124	2.525	0.065	0.064	0.786	0.784	0.689	0.774
M3: PL + SSG + UPSB, FM	3562.115	1126	3.164	0.078	0.088	0.695	0.693	0.609	0.68
M4: PL + SSG + UPSB + FM	4545.136	1127	4.033	0.092	0.081	0.572	0.57	0.502	0.551

Note: PL refers to paradoxical leadership; SSG refers to supervisor−subordinate Guanxi; UPSB refers to unethical pro-supervisor behavior; FM refers to follower mindfulness.

**Table 3:** Mean value, standard deviation, correlation coefficient of variables.

Variables	Me	SD	PL	UPSB	SSG	FM
PL	3.485	0.661	1.000			
UPSB	2.986	0.767	0.291 **	1.000		
SSG	2.993	0.665	0.395 **	0.228 **	1.000	
FM	3.929	0.419	0.188 **	0.076 *	0.133 *	1.000

Note: * refers to *p* < 0.05; ** refers to *p* < 0.01.

**Table 4:** Test of mediating effect of SSG.

Variables	UPSB	UPSB	UPSB	SSG
Control variables				
Gender	−0.144	−0.096	−0.134	−0.069
Age	−0.009	−0.023	0.000	−0.058
Education background	−0.055	−0.062	−0.054	−0.008
Years of work	−0.045	−0.082	−0.055	0.068
Job nature	0.132	0.139	0.129	0.016
Company type	0.006	0.016	0.004	0.011
Employee power distance orientation	0.010	0.013	0.003	0.039
Independent variable				
PL	0.341 ***		0.280 ***	0.407 ***
SSG		0.257 ***		
Mediating variables				
SSG			0.151 ***	
F	0.092 ***	3.690 ***	0.104 ***	0.171 ***
R^2^	0.11	0.078	0.124	0.171
△R^2^	6.131	0.048	6.14	10.281

Note: *** refers to *p* < 0.001.

**Table 5:** Test of moderating effect of FM.

Variables	SSG	SSG	SSG
	M1	M2	M3
Control variables			
Gender	−0.066	0.028	0.021
Age	−0.057	−0.055	−0.057
Education background	−0.005	−0.056	−0.059
Years of work	0.070	0.033	0.031
Job nature	0.017	0.084 *	0.086 *
Company type	0.013	−0.046	−0.049
Employee power distance orientation	0.010	0.004	0.003
Independent variable			
PL	0.404 **	0.146 **	0.172 **
FM		0.624 **	0.632 **
Moderator variables			
FM * PL			0.099 *
F	9.091 ***	36.815 ***	33.889 ***
R^2^	0.173	0.489	0.496
△R^2^	0.173	0.316	0.006

Note: * refers to *p* < 0.05; ** refers to *p* < 0.01; *** refers to *p* < 0.001.

**Table 6:** Bootstrap test result of moderated mediation model.

Mediating Variable	FM	Effect Size	Boot SE	95% Confidence Interval	Index	SE	95% Confidence Interval
SSG	Low (M − SD)	0.074	0.046	[−0.01, 0.173]			
	M	0.081	0.038	[0.017, 0.165]	0.298 ***	0.067	[0.166, 0.431]
	High (M + SD)	0.089	0.039	[0.024, 0.171]			

Note: *** refers to *p* < 0.001.

## Data Availability

The data used to support the findings of this study were obtained from questionnaires and are available upon request from the corresponding author.
